# “Involve Me and I Learn”: Active Learning in a Hybrid Medical Biochemistry First Year Course on an American-Style MD Program in the UAE

**DOI:** 10.1007/s40670-022-01545-6

**Published:** 2022-04-19

**Authors:** Siobhán O’Sullivan, Luciana Aparecida Campos, Ovidiu Constantin Baltatu

**Affiliations:** 1 College of Medicine & Health Sciences, Khalifa University, PO Box 127788, Abu Dhabi, UAE; 2 College of Health Sciences, Abu Dhabi University, Abu Dhabiss, UAE; 3 Center of Innovation, Technology and Education (CITE), Sao Jose dos Campos Technology Park, Sao Jose dos Campos, Brazil; 4 Institute of Biomedical Engineering, Anhembi Morumbi University - Laureate International Universities, Sao Jose dos Campos, Brazil

**Keywords:** COVID-19 teaching, Biochemistry education, Hybrid teaching, Social constructivism, Constructive alignment

## Abstract

Perceived as a subject with abstract jargon, requiring extensive memorization of complex metabolic pathways, chemical structures, and names, students lose sight of the significance of biochemistry on their MD journey (Afshar M, Han Z. Teaching and learning medical biochemistry: Perspectives from a student and an educator. Med Sci Educ. 2014;24:339–41.). A disconnect between what is taught in the classroom and its application to clinical settings arises through over emphasis on the need to pass board exams, documented to be a poor measure of core competencies. Employing active learning strategies with meaningful activities with clinical applications, centered around the curriculum, cognitively engages students and is a deviation from the didactic way in which biochemistry is traditionally taught.

## Background

Basic sciences are the first courses taught to medical students during the preclinical years and often considered the most difficult. Biochemistry is commonly taught in a teacher-centered didactic way. Medical students often have an aversion to biochemistry knowledge as it is seen as a subject with endless facts and jargon, boundless pathways with steps that require short-term rote memorization with little or no connection, and direct relevance to clinical practice. Today’s competitive residency market board examinations (United States Medical Licensing Examination-USMLE, step 1) have become exceedingly important to medical students. Huge emphasis is placed on passing the examination rather than its application to the practice of medicine.

Today’s medical students can be described as millennial or generation Y learners. They expect pedagogical variation, flexibility, speed, and efficiency; are tech savvy; and appreciate being able to control the pace of their learning.

In medical school, with the large amounts of information to be taught and retained by students, there is a tendency to present material in a didactic manner where students are considered empty receptacles that need filling with knowledge passively. If we wish to develop thinking skills and problem-solving abilities, both important lifelong learning skills, a student-centered approach is necessary where the instructor migrates from the “sage on the stage” to the “guide on the side” facilitating and guiding the learning [1].

Active learning activities are associated with better learning outcomes, increased student motivation, satisfaction, engagement in the classroom, and performance [2]. Creative approaches that closely match student learning styles can be used to support the success of all students. As instructors, the impetus is to motivate students, personalize their learning experience, and strive to provide a significant learning experience [3].

The flipped classroom is a popular technology-infused learning model. It has gained popularity in recent years as it shifts learning from the traditional classroom-based didactic lecture to a more dynamic classroom where content is not presented top-down but discussed between peers while facilitated by an instructor [4].

With underpinnings in both social constructivism and active learning, the flipped classroom engages students in content before class, maximizing in-class time for active discussion and engagement. Students take ownership of their learning and are encouraged to reflect on material, analyze, process, and present it, all of which are higher order thinking skills [5]. In addition to arousing curiosity, motivation, and dedication to learning a particular topic, the flipped classroom greatly enhances students’ acquisition and ownership of information and self-directed learning skills [6, 7]. Despite being widely adopted in higher education, systematic reviews of flipped learning have been criticized for lacking a theoretical framework and rigorous evaluation [8, 9]. In review of the literature, however, a number of theoretical frameworks have been identified and proposed including higher order thinking, self-direction, collaboration, problem-based learning, peer-assisted learning, and cognitive load theory, all of which argue for the positive impact of active learning with strong theoretical underpinnings in constructivism [10–12].

Project- and problem-based learning share several characteristics, both being instructional strategies which aim to engage students in authentic tasks to enhance learning. Students work in groups for extended periods of time and are encouraged to seek out information and present it. While both are underpinned by constructivism, the outcomes can differ. Problem-based learning is an inquiry-based approach. A case can be presented framed in a scenario, clues can be identified, and students can use their problem-solving skills to come up with an answer. Students rely on existing knowledge; they search for answers, ask pertinent questions, and often request more information to come up with an answer. The students “learn at their best” when they have “something to care about and get pleasure in being engaged in” [13].

In project-based learning, the end product is summative and this drives the planning. Problem- and project-based learning activities provide instructors with pedagogical latitude to use class time for collaborative exercises that require higher level critical thinking and reasoning skills. Problem- and project-based learning activities are delivered *by* students *for* students, i.e., their peers. They enable students to reach the higher education cognitive outcomes of Bloom’s taxonomy which are fundamentally important in career progression in healthcare professions [14].

Converging project- and problem-based learning with a flipped classroom further promotes Blooms’s higher order learning through promotion of critical thinking, enhanced problem-solving skills, greater student engagement, and collaboration [15].

The skill of the twenty-first century medical graduate will be to articulate the right questions to ask, to navigate through problems, and to understand where and how to search for knowledge. As instructors, the onus is on us to adopt teaching strategies to develop our students’ processes of critical thinking, problem solving, working in a team, delivering a piece of work in a defined period of time, and asking the correct questions all within the context of self-directed learning. Project-based learning enables students to construct knowledge and skills with their own activities [16]. Irrespective of discipline, there has been much research on positive experiences for both student and instructor in project-based, collaborative learning through the development of graduate attributes such as oral and written communication skills as well as confidence and motivation [17, 18]

This study centers on teaching preclinical biochemistry through project-based learning and a flipped classroom approach. Students become teachers in the process, engaging with the curriculum, presenting, and assessing high-stakes topics to their peers through review sessions where theory is presented and contextualized with medical cases. The instructor is on the side observing and steering the process as needed.

## Activity

“MDBS 601 Molecules, Genes and Cells” is the first course taught in medical school in the preclinical curriculum. It covers topics ranging from genetics and molecular biology, anatomy, biomolecules, to metabolism and metabolic disorders. It is a 6-week intensive course written and designed to include a group learning collaborative course learning outcome and assessment component. The course is assessed by in-class quizzes (20%), two large continuous assessments (30%), a group project comprising a presentation and poster (20%) and a final National Board of Medical Education (NBME) style examination (30%). We are a newly established medical school and had enrolled our second cohort of students, 50% of which were international. Approximately, half of the international cohort could not travel to the UAE to start their journey to become medical doctors. As the pandemic worsened, and more restrictions were put in place, these students had huge uncertainty and confusion about their studies, their assessments, and their future. Furthermore, these students had to cope with the sense of isolation having had no opportunity to build and develop personal face-to-face relationships with peers and faculty [19, 20]. The impetus was to build a learning community inclusive of all students, to build peer relationships through group activities while improving the learning experience; a deviation from the traditional didactic way of teaching. Course evaluations from the first cohort had expressed the need for more review sessions on “high-yield” topics and more practice taking and reviewing USMLE style questions. This learning activity was adapted to meet this need.

**Fig. 1:**
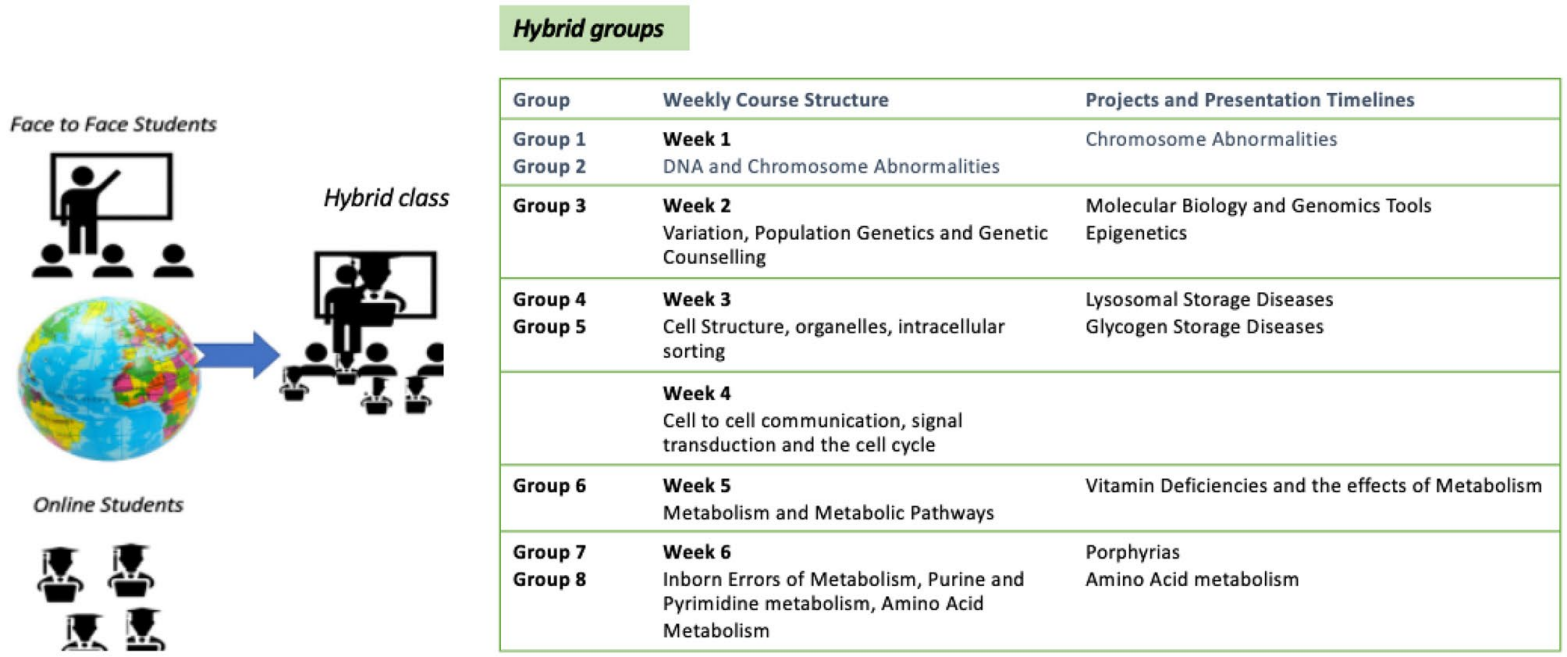
Course structure of “Molecules, Genes and Cells” with the aligned project and presentation timeline

Mixed groups of face-to-face and online students were created and given projects which focused on the USMLE high-stakes topics in medical biochemistry. Topics chosen for each group project aligned with the learning objectives of the course (Fig. 1). For the group project, students were grouped with each group comprising a mixture of face-to-face and online students. Projects were presented to groups and course learning outcomes, and important dates were highlighted for drafts of presentations and posters. Course content in the form of PowerPoint lectures, YouTube videos, lecture videos and sample questions and cases were front-loaded on the learning management system (LMS) and were available for students to review independently. The outline of the course was presented in the initial orientation and each course topic, e.g., week 3: Cell Structure, organelles and intracellular sorting, once taught by the instructor during class time, was subsequently presented as a review session by the group who had that topic to review. Questions and/case studies were presented by the group presenting on the topic (Fig. 1) and class discussions took place with the instructor on the side as the guide.

The outcomes for the collaborative group project were (1) a poster presented to the class on a high-stakes topic, (2) a review session on the topic, and (3) a formative assessment in the form of a question and answer style activity.

Each group had a folder in the LMS where group materials and activities could be stored. These included drafts of posters and presentations, links to discussion boards, resources, and recordings of online meetings. In this way, each of the course learning objectives was aligned with an assessment in the form of a group project comprising a review session and a poster. The poster on the given topic was presented in the review session by the group, and each presentation review ended with questions or cases which were delivered using a range of educational technology tools such as QR codes, Kahoot, and Turning Point technologies so as to include and engage all students in real time. Posters were prepared using PowerPoint or Word and presented as a PDF.

Student review sessions took place on a weekly basis delivered by the group with the assigned topic aligned with the content taught that particular week (Fig. 1). The content of the poster guided the review session with each group member presenting on a different section. This was followed by peer assessment. Some students opted for presenting questions on the screen, allowing time for answering from both online and face-to-face students. While students were given the freedom to design and deliver their own questions/case studies, many opted for computer-mediated learning tools delivered using a range of educational technology tools such as Kahoot and Turning Point technologies so as to include and engage all students in real time. Quizzes were enhanced with images and the leader board on display with real-time feedback of performance-motivated and engaged students [21]. Some students presented the medical application of biochemistry through simple real-life cases.

Subsequent to the review sessions, posters presented on each course topic were enlarged, printed and laminated, and displayed in the students’ learning communities where they served as a learning resource for the entire class. All review session questions were stored in a folder in the LMS for the class to use as a resource.

Students were graded using a rubric which was based on the poster and presentation, first draft outline and final version, the ability to work in a team, and professionalism where students reflect on their work, their contribution, and the contribution of others on their team. Draft and final versions were required by certain dates and all materials were stored in the group folders. Students were encouraged to record their group meetings as evidence that the meetings took place and all recordings were stored in their folder also.

## Results

Two active learning pedagogies, i.e., project-based learning and the flipped classroom (hybrid learning), were integrated into the curriculum with a hybrid class of students (face-to-face and online students), resulting in the production of learning resources and a question and case bank to assist students as they prepare for the end of course exam and a future USMLE exam. Constructive alignment of curriculum content with active learning activities gave students unique opportunities to engage with their learning and understanding of content. Collaborating with each other was even more crucial during the COVID-19 pandemic with students working alone and at a distance from their classmates and classroom.

Hence, each week of the course had review sessions created and delivered by the students to their peers. Embracing flipped classroom pedagogy, various resources were provided to the groups in advance. Embedding active learning strategies in a group work project encouraged self-directed learning and collaborative engagement between the online and face-to-face students.

While scaffolding relationships between online and face-to-face students through learning, online students were further supported through their unexpected first year experience, working online in their home country. In this flipped classroom project-based learning activity, learning activities are delivered and facilitated by students through reflection, analysis, processing, and preparation of materials for discussions with their peers.

## Feedback from Students

Feedback was collected informally in class. The international students said the group work activity made them feel included and part of the class. The online quizzes and cases presented in real time were exciting and engaging. Given that they were not in the classroom with their peers, the interactions and discussions were lively, dynamic, and engaging. Online chat questions were included in all discussions, and questions were directed at them by both the instructor and their peers.

Through informal in-class feedback, oral and written on post-its which students left on their desks, students’ views on the group-based project were collected. They appreciated the opportunity to work in a group and present on a high-stakes topic. They “felt like experts” teaching the topic to their peers and challenging them with questions meant that they had to understand the topic, “to teach it meant I truly had to understand it and to be able to answer questions, I was kept on my toes”.

“I loved that we had the choice to present the questions the way we liked”

For the online students “one thing that I can say helped immensely, was when we were actively involved in the class: When we did the first Kahoot, it really boosted my energy levels and I felt good during that day because for the first time, I felt like a part of the class. Not only that, but I noticed that a lot of the information that was presented on that day was greatly retained”.

“Regarding the flipped classroom session: This approach was so good because not only were we determining the right answer, but we were also reaffirming why the other choices were *incorrect* and that made our learning experience infinitely better.”

“This process of actively involving us in learning the material makes us understand it better, and it also allows us to utilize our independent study time with much *greater efficiency* because we would know our personal areas of difficulty directly from the lecture.”

The comparison to the traditional 50-min lecture was made by several students:

“I think approaches like these are a lot better than traditional lecturing because it is not a constant flow of information with very little time to process what you were presented with, but rather targeted segments of lecturing followed by group discussions to internalize and understand the material.”

“Not only that, but the act of getting us to participate and discuss ramps up our attention and interest in learning the material.”

“Being in a group project helped me connect with my classmates.”

## Key Findings for Medical Education

Technological advances have impacted almost every facet of modern culture and education is no different. With education worldwide being thrown into disarray due to the global COVID-19 pandemic, we as educators have had to broaden our modes of instruction to include online learning to accommodate all students. “Molecules, Genes and Cells” is the first preclinical course MD students take in medical school and is viewed by students as the most difficult. Because it is the first course delivered on the curriculum, students have no choice but to hit the ground running to adjust to the cognitive load and the pace of learning expected of them.

Graduate medical students come from a wide variety of disciplines whether it be the type of degree, the education level, culture, and race. Medical school classes are a kaleidoscope of learners bringing with them diverse prior experiences with various teaching modalities, interests, and life experience. Medical curricula employ compressed coverage of material over limited time frames with little opportunity for repetition, revisitation, or consolidation.

Medicine has long cherished the tradition of the student as the teacher. Throughout the medical education journey, designing strategies that create active learning opportunities for students to assume the role of teacher should be embraced. These include creation of learning venues that encourage interaction, questioning, and debate between peer learners, between learners and teachers, and between learners taking personal responsibility for the discovery of information and dissemination with feedback [22]. In this study, students described themselves as feeling like experts.

“To teach it meant I truly had to understand it and to be able to answer questions, I was kept on my toes”

In this course, students worked together collaboratively and engaged through the discussion boards, creating learning resources which they used to teach and assess their fellow classmates. Working in a group; teaching high-stakes topics; explaining concepts, principles, and processes by putting in their own words; teaching to their peers; justifying answers to questions; and presenting and questioning are empowering for the student and an effective way of measuring understanding [23]. It is the basis of social constructivism, as discovered by Vygotsky in 1978 who proposed that knowledge and higher order thinking occurs first within a social context. In a hybrid class with online and face-to-face students working together in groups, social presence of the students in the online environment is crucial to their engagement with the learning process [24].

Flipped classroom integration in a project enables development of higher order thinking, teamwork, communication, and lifelong learning skills [25]. By designing their poster and presentation and presenting them in the form of a review to their peers and having the flexability to decide on the way they would like to present them, students had choice and also opportunity to demonstrate flair and creativity. They enhanced their communication skills through engaging and challenging interactions with their peers, and had the opportunity to demonstrate adaptability, responsiveness, critical thinking, and self-confidence.

The feedback from the group learning project was encouraging. Our aim was to have a learning community inclusive of all students irrespective of whether they were present in person or virtually. The project, working together in groups, and peer assessing helped students understand key topics in the curriculum through having to teach and assess their peers. The questions and case studies presented are an excellent revision tool for students preparing for the end of course NBME exam and the USMLE which they will take at a later date. Social constructivism and active learning underpin group learning projects. Social collaboration albeit face-to-face or online enables discussions and feedback which can address students’ preconceptions and build on their existing understanding of a topic. While the results of how students performed in their final USMLE exam were not considered or compared to the first cohort, the students did comment on the usefulness of having review sessions for each of the “high-yield” topics and a bank of questions which they could use for revision. Having to work in groups, and prepare the review and questions helped in their understanding of difficult topics. It was a deviation from the traditional lecture, “but rather targeted segments of lecturing followed by group discussions to internalize and understand the material.”

It aligns with a “social constructivist learning paradigm” where learning is defined as a social practice involving a group of students actively participating in a collaborative knowledge construction and understanding process through student to student interaction and building learner communities [26–28].

The purpose of the activity was threefold: to provide a better understanding of the topic, to involve students in their learning through having to teach themselves and each other, and to create a learning environment inclusive of all learners. All three objectives were met.

## Conclusion

Medical students need to learn medically relevant biochemistry. Rote memorization is not synonymous with learning and understanding of material, and the outcome is often learner fatigue. Involving students in the classroom arena through active engagement makes learning more relevant and real. Active learning strategies bring new perspectives and real-world relevance to classroom instruction. Incorporating active learning into the classroom transforms it into a dynamic learning environment with increased student motivation and engagement. Moreover, active learning stimulates higher order thinking, problem solving, and critical analysis that physicians should have, while providing feedback to the instructor. The COVID-19 pandemic has impacted medical education globally and the effects are likely to be long-lasting. Evidently online and hybrid education will become mainstays in the future. Despite the sudden migration of instructional delivery to online platforms, the challenges to faculty and students have been explored and transformed into opportunities; we have had to rethink and redesign the way we teach and assess our students so as to keep them all engaged and give them a sense of belonging [19, 20].

As technology dominates and transforms education, new theories are emerging to explain the learning and teaching framework in the digital age. These are extensions of earlier theories, i.e., social constructivism, behaviorism, and cognitivism which were developed in times when learning was influenced minimally by technology. Social communication increases the impact of learning within the digital age; the digital age and computer-based communication have enabled a rethinking of collaborative learning. Connectivism, put forward by Siemens, is based on social constructivism with technological pedagogy [29, 30].

Advances in medicine and biochemistry are not separate. Significant developments in medicine have been possible through deep understanding of pathological, biochemical, pathogenic, and environmental pathways. Biochemistry teaching of concepts serves as a foundation to understanding the complexities of disease and its management. Marrying two pedagogical platforms as presented in this paper, through authentic experiences and activities, students generate their own learning process; they collaborate, make connections, and develop critical thinking and brain storming through peer discussions and feedback.
